# Effect of prior breast cancer treatment on outcomes of reverse total shoulder arthroplasty

**DOI:** 10.1016/j.jsea.2026.100035

**Published:** 2026-05-07

**Authors:** Loïc Van Oost, Patrick Demey, Henk Loobuyck, Lotte Verstuyft, Hans Van der Bracht

**Affiliations:** a Department of Orthopaedic Surgery, AZ St. Lucas Hospital, Ghent, Belgium; b Department of Gynaecology, AZ St. Lucas Hospital, Ghent, Belgium

**Keywords:** Reverse shoulder arthroplasty, Cuff tear arthropathy, Breast cancer, Radiotherapy, Shoulder surgery, Post-operative complications

## Abstract

**Background:**

Reverse total shoulder arthroplasty (rTSA) provides reliable outcomes in cuff tear arthropathy, but the impact of prior breast cancer treatment on safety and functional outcomes remains unclear.

**Methods:**

We conducted a retrospective cohort study of 15 women with prior ipsilateral breast cancer treatment (mastectomy, lymph node dissection, and/or radiotherapy) who underwent rTSA for cuff tear arthropathy. These results were compared with 50 female patients who underwent rTSA for cuff tear arthropathy without breast cancer history. Outcomes included patient-reported scores (Oxford Shoulder Score, Constant Murley score, and satisfaction), range of motion, pain, and complications, with a minimum follow-up of 2 years.

**Results:**

Oxford Shoulder Score and Constant Murley score were similar between groups (41.5 vs. 41.0; 69.4 vs. 71.8). Satisfaction was high in both cohorts (8.8/10). The breast cancer group showed lower abduction (139° vs. 166°, *P* = .003) and forward flexion (141° vs. 173°, *P* = .003), while internal and external rotation were comparable. Complications were numerically higher in the breast cancer cohort, with revision surgery showing a borderline difference (2/15 vs. 0/50, *P* = .050).

**Conclusion:**

rTSA demonstrated comparable functional outcomes and satisfaction in patients with prior breast cancer treatment. Lower post-operative abduction, forward flexion and a numerically higher complication burden were observed, although complication differences were not statistically significant. Prior breast cancer treatment should not be considered an absolute contraindication, but appropriate pre-operative counseling remains important.

Reverse total shoulder arthroplasty (rTSA) has revolutionized the treatment of complex shoulder conditions including cuff tear arthropathy (CTA), massive cuff tears, osteoarthritis, and rheumatoid arthritis. rTSA has demonstrated favorable functional improvements and a low complication rate, making it a highly effective surgical intervention in the general population.^4^ The use of rTSA in patients with a history of breast cancer, particularly those who underwent mastectomy, axillary lymph node dissection (ALND), or axillary radiation presents specific challenges and areas of uncertainty in clinical practice.^13^

Breast cancer treatments, such as ALND and external beam radiotherapy (XRT), are associated with significant side effects including subcutaneous or muscular fibrosis, lymphedema, recurrent lymphangitis, and tissue necrosis.^13^ These adverse effects can potentially increase the complication rate or impair functional outcomes following shoulder surgery. Consequently, there is reluctance among clinicians to perform shoulder arthroplasty in this patient population.

Several studies have explored the outcomes of shoulder surgery in patients with a history of ALND or XRT, although with varying results. Early research by Andrew et al^2^ identified higher infection rates (10%), increased overall complications, and a substantial incidence of post-operative lymphedema (41%) in patients who underwent mastectomy and ALND prior to shoulder arthroplasty.

Although more recent studies have reported improved outcomes, similar complication rates were still observed.^12^^,^^15^

Beyond ALND, the impact of XRT adds another layer of complexity. Patients with a history of XRT in addition to mastectomy showed similar range of motion (ROM) and visual analog scale pain scores after shoulder surgery compared to a control group.^17^ Marigi et al^13^ concluded that shoulder arthroplasty is effective for improving pain and function in this population, although less favorable compared to patients without prior XRT.

Despite these findings the evidence remains limited, and it is unclear whether upper limb surgery, particularly shoulder arthroplasty, should be routinely considered in patients with a history of breast cancer treatment. The paucity of comprehensive studies underscores the need for further investigation to guide clinical decision-making and optimize patient outcomes. As the number of breast cancer survivors requiring joint replacement continues to grow, it is critical to understand the safety and outcomes of rTSA in this population.

The aim of this study is to evaluate whether a history of breast cancer surgery, including mastectomy with ALND and/or XRT, affects the functional outcomes and complication rates after rTSA for the treatment of CTA. We hypothesized that prior breast cancer treatment would not compromise functional outcomes, although complication rates may be increased.

## Materials and methods

### Patient selection

A retrospective cohort analysis was conducted (level III of evidence) after obtaining approval from the institutional ethics committee. All patients who received rTSA for CTA between December 2016 and May 2023 and who had previous treatment for breast cancer on the same side were identified using electronic medical records. Fifteen patients were detected and were available for further clinical and radiographic evaluation. Tumor type and associated oncological treatments were also reviewed. One patient was excluded in the statistical analysis due to revision to a personalized implant.

Furthermore, a control cohort of 50 consecutive operated female patients treated for CTA with rTSA between 2016 and 2019 was created. The control cohort consisted of 50 consecutive female patients from the same institution and surgeon practice. Controls were not formally matched, but baseline variables including age and body mass index were compared between groups during statistical analysis.

All surgeries in the control and breast cancer cohort were performed by the same surgeon (HVdB) at AZ St. Lucas Hospital, Ghent, Belgium, utilizing a deltopectoral subscapularis-on technique (TM Reverse Zimmer Biomet). All procedures were performed under general anesthesia supplemented with a locoregional plexus block.

Patient-reported outcome measures were assessed using standardized tools, including the Oxford Shoulder Score (OSS), Constant Murley score (CMS), and overall satisfaction of the procedure. General satisfaction was assessed using a self-reported numeric rating scale ranging from 1 (very dissatisfied) to 10 (very satisfied).

These scores were self-reported by the patients and, when necessary, supplemented with data from the electronic medical record or via telephone follow-up. Furthermore, all patients were asked whether they would undergo the same procedure again. Pain was assessed using the pain subscale of the CMS based on a 4-point scale (0, 5, 10, 15), where 0 indicates severe pain and 15 represents the absence of pain.

Complications were analyzed for both groups and included post-operative stiffness, stress fractures, lymphedema, axillary nerve dysfunction, infection, and prolonged post-operative pain, defined as persistent shoulder pain beyond 3 months after surgery requiring additional clinical evaluation or treatment. Persistent post-operative stiffness was defined as a clinically relevant restriction in shoulder motion beyond 3 months after surgery, as judged by the treating surgeon.

All data were anonymized prior to analysis. Potential sources of bias, including selection bias and recall bias, were minimized by cross-referencing patient-reported outcomes with clinical records. A minimal follow-up of 2 years was obtained.

### Statistical analysis

This study employed descriptive and comparative statistical analyses to evaluate the outcomes.

To compare functional outcomes between the 2 groups the CMS, OSS, and ROM measurements (abduction, forward elevation, internal rotation, and external rotation) were analyzed. Data distribution was assessed using the Shapiro–Wilk test. Since the data were not normally distributed, the Mann–Whitney *U* test was applied for continuous variables. For categorical variables, such as complications, a Fisher exact test was used. To adjust for multiple comparisons, the Benjamini-Hochberg false discovery rate correction was applied with a false discovery rate threshold of 0.05.

Absolute ROM values for abduction and forward flexion were extracted directly from the CMS. Internal and external rotation were not measured in degrees but graded on a scale from 0 to 5 to make comparison between groups possible (outlined in Table I). All statistical analyses were conducted using IBM Statistical Package for the Social Sciences software (IBM Corp., Armon, NY, USA), and statistical significance was set at *P* < .05.

**Table I:** Scores for internal and external rotation.

External rotation	Grade
Hand in front of mouth	0
Hand behind head (elbow forward)	1
Hand behind head (elbow back)	2
Hand on top of head (elbow forward)	3
Hand on top of head elbow back	4
Full elevation	5

Internal rotation	Grade
Thigh	0
Buttock	1
Sacroiliac joint	2
Waist	3
T12	4
Shoulder blades	5

## Results

### Patient population

A total of 15 patients were included in the breast cancer cohort and 50 patients in the control cohort. Baseline demographic characteristics of both cohorts are summarized in Tables II and III. In the breast cancer cohort, prior oncologic treatment was heterogeneous. Surgical treatment included tumorectomy (10/15) and mastectomy (5/15), often combined with sentinel node procedures or axillary dissection. Radiotherapy (RT) (9/15) was the most common adjuvant therapy, followed by chemotherapy (4/15). Histopathological tumor types included ductal carcinoma in situ (DCIS, n = 4), invasive lobular carcinoma (ILC, n = 2), and invasive ductal carcinoma (IDC, n = 8). One patient did not have a documented tumor type.

**Table II:** Demographic data.

Characteristic	Breast cancer cohort (n = 14)	Control cohort (n = 50)	*P* value
Age (yr)	75.07 (±5.17)	71.82 (±7.26)	.164
BMI (kg/m^2^)	26.67 (±4.56)	28.58 (±5.11)	.180

*BMI*, body mass index.

Values are presented as mean ± standard deviation.

**Table III:** Demographics, oncologic treatment history, and post-operative complications in breast cancer cohort.

Patient ID	Age (yr)	BMI	Cancer type	Treatment modalities						Complications
				T	M	S	A	RT	CT	
1	80	27.6	DCIS	x						Acromial stress fracture
2	69	33.9	N/A	x						
3	70	33.8	ILC	x		x		x	x	
4	69	25.0	DCIS	x		x		x		
5	84	25.2	IDC	x		x		x	x	
6	72	23.1	IDC		x		x	x	x	
7	68	21.9	IDC		x			x	x	
8	74	27.07	DCIS	x						
9	83	33.01	ILC		x					
10	78	30.8	IDC	x				x		
11	76	23.44	IDC	x				x		
12	78	22.58	IDC	x				x		
13∗	75	27.16	IDC		x		x			Glenoidal fracture with revision to a custom-made implant postoperative stiffness, lymphedema
14	74	25.71	IDC	x		x		x		
15	76	20.27	DCIS		x			x	x	Post-operative stiffness with reintervention for arthrolysis

*BMI*, body mass index; *DCIS*, ductcarcinoma in situ; *ILC*, invasive lobular carcinoma; *IDC*, Invasive duct carcinoma; *T*, tumorectomy; *S*, sentinel procedure; *RT*, radiotherapy; *CT*, chemotherapy; *M*, mastectomy; *A*, axillary node dissection.

∗ Not included in statistical analysis due to revision to patient specific implant.

### Complications

Complications were categorized as either major or minor based on clinical severity and the need for surgical intervention. Major complications included any events requiring a return to the operating room. This comprised revision surgery, such as a two-stage conversion to a custom-made implant, as well as severe post-operative stiffness necessitating surgical arthrolysis. Minor complications were defined as events that resolved with nonoperative management, including mild stiffness, conservatively treated stress fracture, and lymphedema.

Three patients with complications were reported in the breast cancer cohort, comprising 2 patients with major complications (glenoid fracture and persisting stiffness) and 1 patient with minor complications (conservatively treated acromial stress fracture). Patient 1 (breast-conserving surgery) developed an acromial stress fracture 38 months after surgery. Radiographs demonstrated a spine fracture with limited displacement, classified as Levy type 2. The lesion was managed conservatively. Patient 13 (mastectomy with ALND) sustained an atraumatic periprosthetic glenoid fracture seven months post-operatively, which required a two-stage revision with a custom-made implant. The same patient also developed post-operative stiffness and lymphedema following the revision surgery. Patient 15 (mastectomy with RT and chemotherapy) developed post-operative stiffness necessitating open arthrolysis ten months after the index procedure. Multiple intraoperative tissue cultures did not confirm infection. Functional outcomes were assessed after this secondary intervention.

When comparing both cohorts, revision surgery was required in 2 patients (13.3%) from the breast cancer cohort and in none of the controls (*P* = .050).

Shoulder stiffness was observed in 1 patient (6.66%) in the breast cancer group and in none of the controls (*P* = .231).

Regarding fractures, 1 acromial stress fracture and 1 atraumatic periprosthetic glenoid fracture were identified in the breast cancer cohort. Overall, atraumatic fractures occurred in 2 patients (13.3%) compared with 2 patients (4.0%) in the control cohort (*P* = .226).

No dislocations were reported (Table IV). All patients had a minimum follow-up of two years, with a mean duration of 61.3 months (5.1 years).

**Table IV:** Post-operative complications observed in both groups.

Complication	Breast cancer cohort (n = 15)	Control cohort (n = 50)	*P* value
Revision surgery	2 (13.3%)	0 (0%)	.050
Dislocation	0 (0%)	0 (0%)	1.000
Atraumatic or stress fracture	2 (13.3%)	2 (4.0%)	.226
Surgical site infection	0 (0%)	0 (0%)	1.000
Stiffness	1 (6.66%)	0 (0%)	.231

*P* values calculated using Fisher exact test (2-sided), statistically significant at *P* < .05.

### Functional outcomes

The clinical outcomes of the breast cancer cohort and control cohort are presented in Table V. One patient (patient 13) was excluded from the functional scores due to revision to a personalized implant, although these results are reported separately.

**Table V:** Functional results based on OSS and CMS.

Variable	Breast cancer cohort Mean (±SD) n = 14	Control cohort Mean (±SD) n = 50	*P* value	Adjusted *P* value
Functional scores				
OSS(points)	41.50 (±7.42)	41.04 (±9.38)	.578	.809
CMS (points)	69.36 (±18.96)	71.76 (±14.16)	.696	.812
Range of motion				
Abduction (°)	139.29 (±34.52)	166.20 (±29.82)	<.001	.003∗
Forward flexion (°)	141.43 (±29.83)	172.80 (±18.74)	<.001	.003∗
Internal rotation (score)	2.64 (±1.34)	2.60 (±1.47)	.907	.907
External rotation (score)	3.86 (±1.10)	3.86 (±0.76)	.564	.809
Pain (points)	13.57 (±3.06)	12.80 (±3.66)	.497	.809

*OSS*, Oxford Shoulder Score; *CMS*, Constant Murley score; *SD*, standard deviation; *FDR*, false discovery rate.

Values are expressed as mean ± standard deviation.

*P* values were calculated using the Mann–Whitney *U* test.

∗ Adjusted *P* values are statistically significant after Benjamini–Hochberg correction (FDR, threshold = 0.05).

### Functional scores

**Figure 1:**
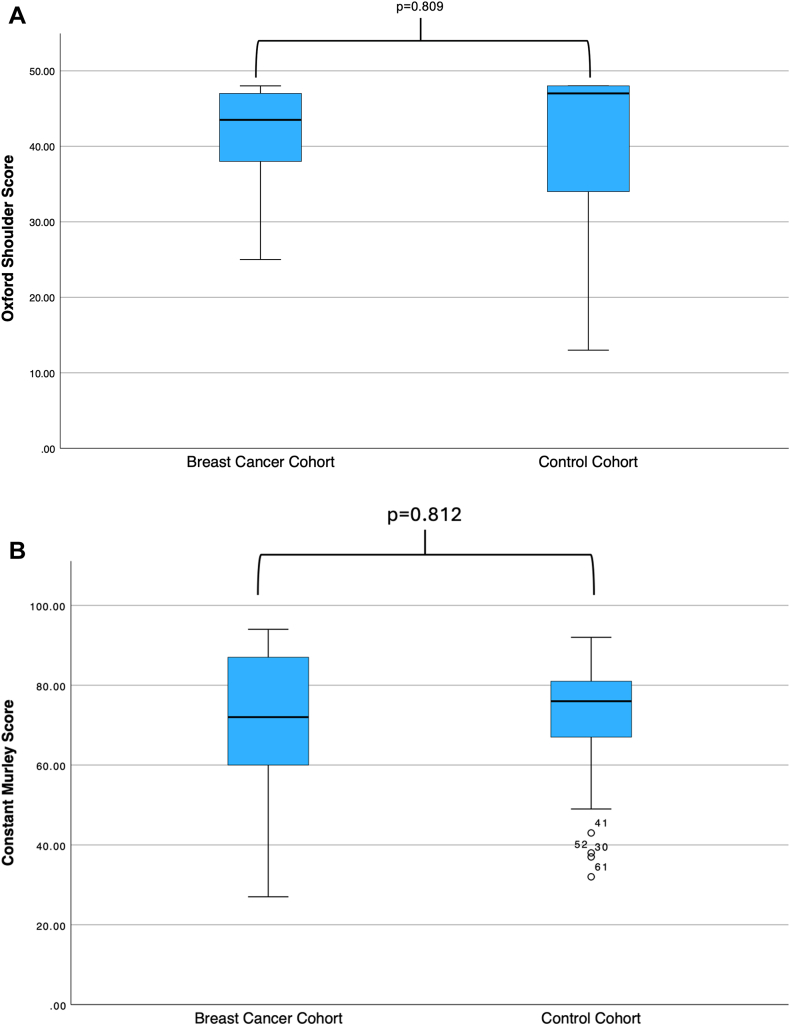
Comparison of OSS (**A**) and CMS (**B**) between the breast cancer and control cohorts. No significant differences were found (*P* = .809 and *P* = .812, respectively). *OSS*, Oxford Shoulder Score; *CMS*, Constant Murley score.

Both the OSS (41.50 ± 7.42 vs. 41.04 ± 9.38; *P* = .809) and the CMS (69.36 ± 18.96 vs. 71.76 ± 14.16; *P* = .812) showed no significant differences between the groups, indicating comparable shoulder functionality (Fig. 1).

### Range of motion

Significant differences in ROM were observed between the groups for abduction (139.29° ± 34.52 vs. 166.20° ± 29.82; *P* = .003) and forward flexion (141.43° ± 29.83 vs. 172.80° ± 18.74; *P* = .003), with the control cohort demonstrating superior outcomes. No significant differences were found for internal rotation (2.64 ± 1.34 vs. 2.60 ± 1.47; *P* = .907) or external rotation (3.86 ± 1.10 vs. 3.86 ± 0.76; *P* = .809).

### Pain

The mean pain score in the breast cancer cohort was 13.57 ± 3.06, while in the control cohort it was 12.80 ± 3.66. Although a small difference in mean scores was observed, this difference was not statistically significant (*P* = .809).

### Patient satisfaction

In the breast cancer cohort, 81% of patients reported that they would opt for the same procedure again. The breast cancer cohort reported a mean satisfaction score of 8.77/10, while the control cohort reported a mean score of 8.86/10. Both the breast cancer cohort and control cohort had similar very satisfying results.

## Discussion

The use of rTSA has grown substantially in recent years due to its proven efficacy in improving shoulder function and patient outcomes, particularly in cases of rotator cuff-deficient shoulders.^7^

Simultaneously, the global incidence of breast cancer has risen significantly with advancements in oncological treatments contributing to improved survival rates.^3^ This has led to an increasing number of breast cancer survivors; therefore, understanding the impact of oncological treatments on subsequent orthopedic surgeries is crucial for improving patient outcomes.

Patients with a history of breast cancer, similar to the general population, may develop shoulder joint degeneration that requires total shoulder prosthesis. However, these patients often face unique challenges during and after shoulder surgery. Previous treatments, including RT and lymph node dissection, are associated with increased risks of post-operative complications, such as impaired wound healing, infection, and reduced functional recovery.^14^

These complications may be due to radiation-induced fibrosis, lymphatic obstruction, and reduced vascularization in the affected tissues.^8^

These factors have led to a general hesitancy to perform shoulder surgery, including rTSA, in this population. Despite this, limited data exist on the outcomes of rTSA in breast cancer survivors, making it a critical area for further research.

To our knowledge, this is the first comparative study evaluating an isolated cohort of patients undergoing rTSA for CTA after prior breast cancer treatment vs. a control cohort. Our analysis revealed several important findings and suggests that prior breast cancer treatment should not automatically be considered a contraindication for rTSA.

## Complications

Overall, 3 out of 15 patients (20%) experienced at least one complication. In the control cohort, only 2 patients (4.0%) experienced a complication. Although numerically higher, this difference was not statistically significant.

Two major complications were documented in the breast cancer cohort. One patient experienced a major complication requiring revision surgery with a two-stage conversion to a custom-made implant and another required surgical arthrolysis due to severe post-operative stiffness. In addition to the 2 major complications, 1 patient developed a stress fracture, which was managed nonoperatively.

No differences in post-operative instability were observed, as no dislocations occurred. This finding is consistent with the results reported by Lee et al.^12^

Previous studies often highlight a concern regarding post-operative lymphedema. However, in this study population, this was not a major issue. None of the patients with a primary rTSA developed lymphedema. Only the one patient with a glenoid fracture and previous invasive ductal carcinoma (treated with mastectomy, ALND, and hormone therapy) developed lymphedema after revision surgery. Lymph node dissection is widely acknowledged as a major risk factor for the development of lymphedema.^9^

In this study, only 2 patients underwent lymph node dissection as part of their treatment. This may explain why only one case of post-operative lymphedema was observed. The presence of pre-existing lymphedema is a notable concern when performing surgery. In a study by Lee et al,^12^ shoulder arthroplasty in patients with existing lymphedema was associated with a high complication rate of 21%, including a 16% infections.

No infections were observed in this study, which contrasts with the existing literature. Andrews et al^2^ identified an increased post-operative infection risk in breast cancer survivors.

### Patient satisfaction

The findings of our study with mean satisfaction scores of 8.77/10 and 8.86/10 in the breast cancer and control cohorts, respectively, show great perceived satisfaction rates in both groups. Furthermore, 81% of the breast cancer cohort indicated that they would choose to undergo the procedure again. These findings align well with previously published orthopedic literature. In a large cohort of healthy patients undergoing rTSA, 94% reported they would opt for the same procedure again, and 82% was satisfied or very satisfied with the procedure.^16^

Despite prior oncological history, patients in the breast cancer cohort reported similarly high satisfaction rates compared to controls.

### Functional evaluation

The breast cancer cohort demonstrated overall satisfaction with the procedure, as reflected in the functional scores (CMS and OSS). Even when comparing with a cohort, no differences in functional outcomes are observed regarding patient-reported outcome measures.

### Range of motion

A significant difference was observed between the 2 groups in terms of abduction and elevation. However, no significant differences were noted for internal and external rotation. Interpretation of post-operative ROM differences warrants caution. Pre-operative shoulder motion was not consistently available, and post-operative motion is known to be influenced by baseline functional status.^6^ Therefore, the lower post-operative abduction and forward flexion observed in the breast cancer cohort cannot be attributed solely to prior oncologic history or treatment.

The clinical outcomes of patients who developed minor complications, such as post-operative stiffness, were included in the overall analysis. Patients with these minor complications tended to perform worse compared with those without complications. As the breast cancer cohort included a higher proportion of patients with minor complications relative to the control group, this may partly account for the lower mean clinical outcomes observed in this subgroup. This is consistent with the results reported by Marigi et al.^13^

It is important to note that despite the reduced ROM, these patients do not perceive it as such, as they consistently achieve high scores on the CMS and OSS score.

### Pain

Overall pain levels were low in both cohorts, with a mean score of 13.57 in the breast cancer cohort and 12.80 in the control cohort, suggesting a generally favorable pain outcome. Pain-related outcomes in breast cancer survivors remain a recurrent concern in the current literature. Previous studies have shown that patients with a history of breast cancer tend to report more pronounced post-operative pain and less favorable pain-related outcomes following shoulder surgery.^1^ In contrast, our study revealed low pain scores in both the breast cancer and control cohort.

In addition to advancements in shoulder surgery, improvements in breast cancer surgery may likewise have contributed to the enhanced clinical and functional outcomes in the breast cancer cohort. There is an improved screening for breast cancer, as well as an increase in the use of breast-conserving surgery. In the past, a full lymph node dissection was performed. The introduction of sentinel lymph node biopsy has become the gold standard, leading to a reduction in the number of complete lymph node dissections for node-negative disease.^5^

In addition, the development of the core needle biopsy introduced a significantly less invasive procedure for treatment as it allows clinicians to decide on performing a tumorectomy instead of a full mastectomy.^18^ These technological advancements have reduced side effects, potentially lowering the threshold for subsequent surgical interventions.

An important consideration is that the reduction in bone density is further exacerbated by hormone therapy commonly prescribed following breast cancer surgery. This therapy often includes the use of aromatase inhibitors, which are known to significantly decrease bone density.^10^ Although the increased risk may be subtle, our findings illustrate a relevant concern, as demonstrated by a glenoid fracture in a patient undergoing long-term treatment with an aromatase inhibitor after the breast cancer surgery.

Our findings suggest a trend toward higher rates of specific post-operative complications, including stiffness and revision surgery, in patients with a history of breast cancer. However, these differences were not statistically significant. Despite the higher overall complication rate in this cohort, functional outcomes and patient satisfaction were not adversely affected. Moreover, these patients demonstrate a similar prevalence of advanced degenerative shoulder conditions, such as CTA or omarthrosis, compared to the general population and often present with significant pain.^11^ Consequently, surgical intervention should not be withheld solely due to an oncologic history.

## Limitations

This study has several limitations. First, its retrospective design may have introduced selection and information bias. Second, the relatively small sample size limits statistical power and increases the risk of type II error. Third, post-operative stiffness was assessed by an experienced clinician but remains partly dependent on clinical judgment. Furthermore, no standardized in-person clinical evaluation was performed at the time of questionnaire completion or telephone follow-up, which may have increased the influence of patient perception on reported outcomes. Pre-operative ROM was not consistently documented in the medical records, limiting the ability to fully assess baseline functional differences between groups. In addition, the control cohort was not formally matched and residual confounding cannot be excluded. Additional medical comorbidities, including diabetes, endocrine disorders, and overall comorbidity burden, were not consistently documented and therefore could not be fully adjusted for in the analysis. An additional limitation is the heterogeneity of prior breast cancer treatment within the study cohort. Treatment-related factors such as mastectomy, axillary surgery, and RT may have influenced post-operative outcomes and complication risk. Meaningful subgroup analyses were not feasible because of the limited sample size.

## Conclusion

Advancements in surgical techniques in both orthopedic surgery and breast cancer surgery have significantly reduced the incidence of adverse effects. Patients reported high post-operative satisfaction despite significantly lower ROM. A numerically higher complication burden was observed in the breast cancer cohort, although no statistically significant differences were detected.

These findings suggest that a history of breast cancer should not automatically be considered a contraindication for rTSA. Careful pre-operative assessment remains important, particularly in patients with prior oncologic treatment. Patients should be counseled that post-operative complications may occur, although functional outcomes and satisfaction remained favorable in this cohort. Further research with larger cohorts and prospective study designs is needed to clarify the influence of specific treatment modalities and optimize surgical decision-making in this patient population.

## Declaration of generative AI and AI-assisted technologies in the writing process

During the preparation of this work the authors used Open AI in order to improve readability and language. After using this tool, the authors reviewed and edited the content as needed and take full responsibility for the content of the publication.

## Disclaimers:

Funding: No funding was disclosed by the authors.

Conflicts of interest: The authors, their immediate families, and any research foundations with which they are affiliated have not received any financial payments or other benefits from any commercial entity related to the subject of this article.

## References

[1] Al-Hilli Z., Wilkerson A. (2021). Breast surgery: Management postoperative complications following operations breast cancer. Surg Clin North Am.

[2] Andrews L.R., Cofield R.H., O’Driscoll S.W. (2000). Shoulder arthroplasty in patients with prior mastectomy for breast cancer. J Shoulder Elbow Surg.

[3] Bray F., Ferlay J., Soerjomataram I., Siegel R.L., Torre L.A., Jemal A. (2018). Global cancer statistics 2018: GLOBOCAN estimates of incidence and mortality worldwide for 36 cancers in 185 countries. CA Cancer J Clin.

[4] Burden E.G., Batten T.J., Smith C.D., Evans J.P. (2021). Reverse total shoulder arthroplasty: a systematic review and meta-analysis of complications and patient outcomes dependent on prosthesis design. Bone Joint J.

[5] Cheifetz R., McKevitt E. (2023). Advances in the surgical treatment of breast cancer. Curr Oncol.

[6] Crutsen J.R.W., Lambers Heerspink F.O., van Leent E.A.P., Janssen E.R.C. (2024). Predictive factors for postoperative outcomes after reverse shoulder arthroplasty: a systematic review. BMC Musculoskelet Disord.

[7] Day J.S., Lau E., Ong K.L., Williams G.R., Ramsey M.L., Kurtz S.M. (2010). Prevalence and projections of total shoulder and elbow arthroplasty in the United States to 2015. J Shoulder Elbow Surg.

[8] Delanian S., Lefaix J.L., Pradat P.F. (2012). Radiation-induced neuropathy in cancer survivors. Radiother Oncol.

[9] Donahue P.M.C., MacKenzie A., Filipovic A., Koelmeyer L. (2023). Advances in the prevention and treatment of breast cancer-related lymphedema. Breast Cancer Res Treat.

[10] Hamood R., Hamood H., Merhasin I., Keinan-Boker L. (2019). Hormone therapy and osteoporosis in breast cancer survivors: assessment of risk and adherence to screening recommendations. Osteoporos Int.

[11] Klarić-Kukuz I., Aljinović J., Barun B., Roki M., Benzon B., Budimir Mršić D. (2025). Subacromial pain syndrome in breast cancer Survivors—Are structural shoulder changes verified by ultrasound clinically relevant?. Diagnostics.

[12] Lee J., Nguyen N.T.V., Shukla D., Sperling J.W., Cofield R.H., Sanchez-Sotelo J. (2020). Shoulder arthroplasty in patients with upper extremity lymphedema may result in transient or permanent lymphedema worsening. Shoulder Elbow.

[13] Marigi E.M., Johnson Q.J., Dancy M.E., Barlow J.D., Crowe M.M., Sperling J.W. (2023). Shoulder arthroplasty after prior external beam radiation therapy: a matched cohort analysis. J Shoulder Elbow Surg.

[14] McNeely M.L., Campbell K.L., Rowe B.H., Klassen T.P., Mackey J.R., Courneya K.S. (2006). Effects of exercise on breast cancer patients and survivors: a systematic review and meta-analysis. CMAJ.

[15] Padegimas E.M., Merkow D., Nicholson T.A., Lazarus M.D., Ramsey M.L., Williams G.R. (2019). Outcomes of shoulder arthroplasty following axillary lymph node dissection. Shoulder Elbow.

[16] Sheth M.M., Heldt B.L., Spell J.H., Vidal E.A., Laughlin M.S., Morris B.J. (2022). Patient satisfaction and clinical outcomes of reverse shoulder arthroplasty: a minimum of 10 years’ follow-up. J Shoulder Elbow Surg.

[17] Taylor K.A., Warren J.R., Jildeh T.R., Kuhlmann N., Pietroski A.D., Beydoun R. (2022). The impact of external beam radiation therapy on shoulder surgical outcomes: a case series study. J Shoulder Elbow Surg.

[18] Wyld L., Audisio R.A., Poston G.J. (2015). The evolution of cancer surgery and future perspectives. Nat Rev Clin Oncol.

